# Clinical value of anoikis-related genes and molecular subtypes identification in bladder urothelial carcinoma and *in vitro* validation

**DOI:** 10.3389/fimmu.2023.1122570

**Published:** 2023-05-18

**Authors:** Ying Dong, Chaojie Xu, Ganglin Su, Yanfeng Li, Bing Yan, Yuhan Liu, Tao Yin, Shuanzhu Mou, Hongbing Mei

**Affiliations:** ^1^ Department of Urology, The First Affiliated Hospital of Shenzhen University, Shenzhen Second People's Hospital, Shenzhen University, Shenzhen, China; ^2^ Key Laboratory of Medical Reprogramming Technology, The First Affiliated Hospital of Shenzhen University, Shenzhen Second People's Hospital, Shenzhen, China; ^3^ Guangdong Key Laboratory of Systems Biology and Synthetic Biology for Urogenital Tumors, The First Affiliated Hospital of Shenzhen University, Shenzhen Second People's Hospital, Shenzhen, China; ^4^ Department of Urology, Peking University First Hospital, Institution of Urology, Peking University, Beijing Key Laboratory of Urogenital Diseases (Male) Molecular Diagnosis and Treatment Center, National Urological Cancer Center, Beijing, China; ^5^ Department of Urology, Peking University First Hospital, Beijing, China

**Keywords:** bladder urothelial carcinoma, anoikis, tumor microenvironment, risk score, immunotherapy

## Abstract

**Background:**

Anoikis is a programmed cell death process that was proven to be associated with cancer. Uroepithelial carcinoma of the bladder (BLCA) is a malignant disease of the urinary tract and has a strong metastatic potential. To determine whether anoikis-associated genes can predict the prognosis of BLCA accurately, we evaluated the prognostic value of anoikis-associated genes in BLCA and constructed the best model to predict prognosis.

**Method:**

The BLCA transcriptome data were downloaded from TCGA and GEO databases, and genes with differential expression were selected and then clustered using non-negative matrix factorization (NMF). The genes with the most correlation with anoikis were screened and identified using univariate Cox regression, lasso regression, and multivariate Cox regression. The GEO dataset was used for external validation. Nomograms were created based on risk characteristics in combination with clinical variants and the performance of the model was validated with receiver operating characteristic (ROC) curves. The immunotherapeutic significance of this risk score was assessed using the immune phenomenon score (IPS). IC50 values of predictive chemotherapeutic agents were calculated. Finally, we used RT-qPCR to determine the mRNA expression of four genes, *CALR*, *FASN*, *CASP6*, and *RAD9A*.

**Result:**

We screened 406 tumor samples and 19 normal tissue samples from the TCGA database. Based on anoikis-associated genes, we classified patients into two subtypes (C1 and C2) using NMF method. Subsequently, nine core genes were screened by multiple methods after analysis, which were used to construct risk profiles. The design of nomograms based on risk profiles and clinical variables, ROC, and calibration curves confirmed that the model could well have the ability to predict the survival of BLCA patients at 1, 3, and 5 years. By predicting the IC50 values of chemotherapeutic drugs, it was learned that the high-risk group (HRG) was more susceptible to paclitaxel, gemcitabine, and cisplatin, and the low-risk group (LRG) was more susceptible to veriparib and afatinib.

**Conclusion:**

In summary, the risk score of anoikis-associated genes can be applied as a predictor to predict the prognosis of BLCA in clinical practice.

## 1 Introduction

Bladder cancer is one of the most malignant tumors of the urinary tract with metastatic potential and a high annual morbidity and mortality rate (1). Approximately 75% of patients with bladder cancer present with tumors confined to the mucosa or submucosa (Ta, CIS, or T1 stage); this percentage is even higher in patients under the age of forty (2). The most predominant type of this pathological cell is uroepithelial carcinoma, which accounts for about 90% of cases (3). While approximately 50% of patients present with tumors confined to the mucosa or submucosa (4), the clinical features of bladder urothelial carcinoma (BLCA) are complex and the early symptoms are not typical (5), so a significant proportion (72%) are diagnosed at a late stage, when the tumor has already metastasized, resulting in poor treatment outcomes (6). Therefore, there is a critical need to develop reliable predictors of treatment response and prognosis to improve individualized care”.

Anoikis is a form of programmed cell death that occurs when cells lose contact with the extracellular matrix (7). It is a critical mechanism for maintaining tissue integrity by preventing unattached cells from dividing, growing, or attaching to an unsuitable matrix. Anoikis resistance is associated with tumor progression and metastasis, as cancer cells are able to detach and survive without undergoing cell death, allowing them to colonize distant organs (8, 9). Thus, the ability to detect and predict anoikis resistance in bladder cancer patients could be an important tool for predicting disease progression and improving treatment outcomes.

In this study, we investigated the role of anoikis-related genes in bladder cancer using data from the GEO and TCGA-BLCA cohorts. Specifically, we aimed to identify a set of genes associated with anoikis resistance that could be used to predict treatment response and prognosis. We also examined the potential signaling pathways involved in anoikis resistance and assessed the sensitivity of different chemotherapeutic drugs in relation to the risk score generated from our gene expression analysis. By addressing these questions, we hope to provide important insights into the prognostic value of anoikis-related genes in bladder cancer and highlight potential therapeutic targets for the disease.

## 2 Materials and methods

### 2.1 Collection of multi-omics data

Pathological samples related to BLCA and normal bladder tissue data were obtained from GSE31684 cohort and TCGA-BLCA dataset. After removing patients with no clinical information, 93 tumor samples were obtained in GSE31684. 406 cases of tumors and 19 cases of normal samples were selected from the TCGA-BLCA cohort. The transcriptome profiles of these samples were retrieved, and then 434 potential genes associated with anoikis were screened through the literature and Genecards database (**Table S1**). We identified the FDR (false discovery rate)< 0.05 and |log2Fold Change (FC)|>1 as thresholds for screening differentially expressed genes (DEGs).

### 2.2 Cluster typing by non-negative matrix factorization method

NMF was used instead of hierarchical clustering for tumor typing (10). The biological correlation coefficients were extracted using the R package “NMF” algorithm to cluster the tumor samples with internal features (11). The survival curves of the C1 and C2 categories and their correlation with the traditional tumor microenvironment classification groups were analyzed and compared.

### 2.3 Landscape of tumor microenvironment infiltrating cells

The subtype abundance of 9 different immune cells and stromal cells that could represent the tumor immune microenvironment was obtained. Based on the microenvironment cell populations, infiltrating cells were identified by comparing scores between the two groups.

### 2.4 Signature was constructed based on anoikis -related genes

After using univariate Cox regression, genes associated with anoikis were identified. They were processed by lasso regression analysis and multivariate Cox regression analysis to prevent overfitting of these genes, and at the same time analyzing the prognostic characteristics of these genes, the risk score of each sample was determined using the formula equation:


$$riskscore=\sum_{i=1}^{n}\left(coefi*Xi\right)$$

Here, coef was the regression coefficient in the multivariate Cox regression analysis as described previously. X indicated the expression of candidate genes. And the median risk score was used as the cut-off point, each sample was divided into high-risk group (HRG) and low-risk group (LRG), and the overall survival rate of the HRG and LRG was analyzed. The survival curves of the HRG and LRG of the related genes screened were analyzed separately.

### 2.5 Establishment and verification of the nomogram

Receiver operating characteristic (ROC) analysis was performed on risk scores and clinical variables at years 1, 3, and 5 to determine the best prognostic indicators. The R program “rms” was established and then “regplot” was used to complete the visualization. The calibration curve was used to evaluate the consistency of the model.

### 2.6 Functional enrichment of anoikis-associated genes

Gene set enrichment analysis (GAES) was used to label the gene function of the HRG and LRG, and the first 8 results were visualized, which were selected with the results’ statistically significant (P<0.05).

### 2.7 Correlation of risk characteristics and clinical variables

The distribution of clinical variables in HRG and LRG was visualized, and the proportion of patients in the subgroups of clinical variables was displayed.

### 2.8 Collection and processing of epigenetic mutation data

Information on the corresponding somatic alterations was obtained from TCGA-BLCA. The 20 most common driver genes with frequent somatic mutations were presented. Based on the risk of TMB and the HRG and LRG of the samples, patients are divided into four major categories, and their survival rates are calculated separately to construct survival curves (12).

### 2.9 Correlation of risk scores with immune infiltrating cells

We used XCELL, TIMER, QUANTISEQ, MCP COUNTER, EPIC, CIBERSORT, and CIBERSORT-ABS to reveal the correlation between risk score and immune infiltrating cells (13).

### 2.10 Gene set variation analysis

The pathway of the core gene was analyzed using the KEGG database to assess the degree of activation of its signature pathway and metabolic pathways. Normalized GSVA was calculated for each gene set in each sample to determine the relative pathway activity, immune markers, and immune checkpoints for each gene set.

### 2.11 Predicting patient response to immunotherapy and chemotherapy drug therapy

After obtaining immune checkpoint blockage-associated genes, their expression levels were explored according to the risk score. Immunophenoscore (IPS) uses the expression of genes associated with immune checkpoints to assess the immunogenicity of tumors in high- and low-risk samples.

By constructing a cell expression family tree based on TCGA-BLCA data. For tumor drug susceptibility genomics, the R package “pRRophetic” was used to predict the sensitivity of tissues to different drugs.

### 2.12 Experimental verification

Four cell lines, UMUC3, RT112, T24, and SV-HUC1, were selected. Cells were cultured in MEM, RPMI-1640, 5A, and F-12K medium plus 10% fetal bovine serum, respectively, at 37°C in a 5% CO_2_ environment. The four different cell lines were subjected to real-time quantitative polymerase chain reaction (RT-qPCR), and the relative expression of *CALR, CASP6, FASN*, and *RAD9A* was calculated using the 2-ΔΔ^Ct^ method with glyceraldehyde-3-phosphate dehydrogenase (GAPDH) levels as the endogenous control. The primer sequences used for PCR were as follows: :*CALR*: F: CCTGCCGTCTACTTCAAGGAG R: GAACTTGCCGGAACTGAGAAC; *CASP6*: F: ATGGCGAAGGCAATCACATTT R: GTGCTGGTTTCCCCGACAT; *FASN*: F: AAGGACCTGTCTAGGTTTGATGC R: TGGCTTCATAGGTGACTTCCA; *RAD9A*: F: CATTGACTCTTACATGATCGCCA R: GCCAGGTGAAAGGGAAATGG.

### 2.13 Statistical analysis

The Kruskal-Wallis test was used to compare data from more than two groups, and Wilcoxon was used to compare data from two groups. Kaplan-Meier log-rank test was used to evaluate each survival curve. A chi-square test was performed to correlate risk score subgroups with somatic mutation frequency, and Spearman analysis was performed to calculate the correlation coefficient. The results of the CIBERSORT algorithm with p< 0.05 were used for further analysis. p< 0.05 was considered statistically significant. R software was used for all statistical analyses.

## 3 Results

### 3.1 Removing batch effects and normalizing data

**Figure 1:**
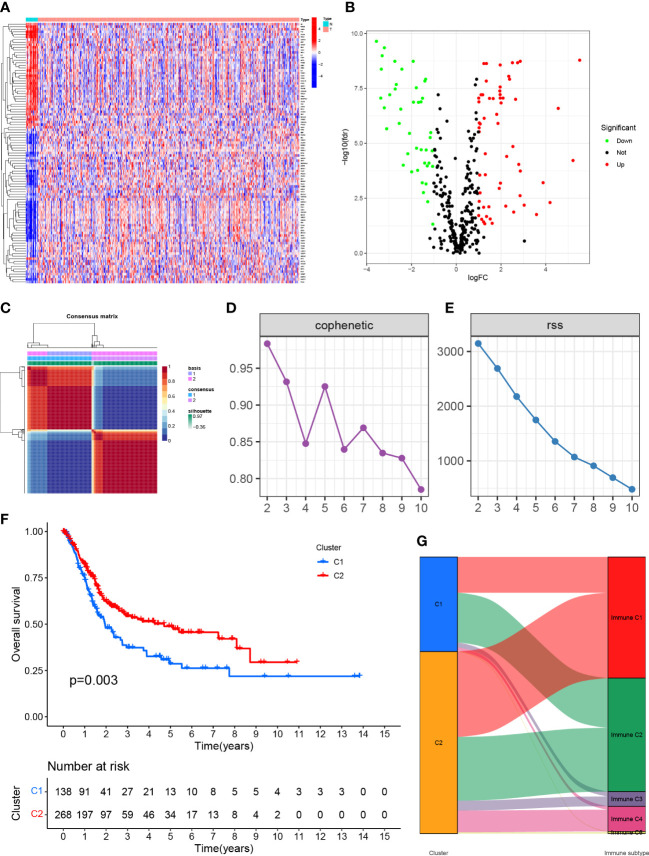
**(A)** Expression of genes associated with anoikis in tissues in different samples. **(B)** Volcano map of differentially expressed genes. **(C)** Mapping clustering based on NMF algorithm. **(D, E)** Multiple ways to determine cluster-related performance and stability. **(F)** C1 and C2 had significant differences in overall survival. **(G)** Proportion of C1 and C2 in immune molecule subtypes.

We obtained 406 tumor tissue samples with clinical information and 19 normal tissue samples based on TCGA-BLCA data. The Clinicopathological characteristics of BLCA patients from the TCGA and GEO databases were shown as **Table S1**. From the **Table S1**, we can know that there are 160 patients are equal to or over 65 years old,131 patients are at stage I-II and ect. After removal of batch effects, normalization was performed. We obtained 101 DEGs (**Figure 1A**; **Table S2**), and the top 50 genes that could express differentially were shown using a heatmap (**Figure 1B**).

### 3.2 NMF was used to analyze the molecular subtypes of anoikis-associated genes

**Figure 2:**
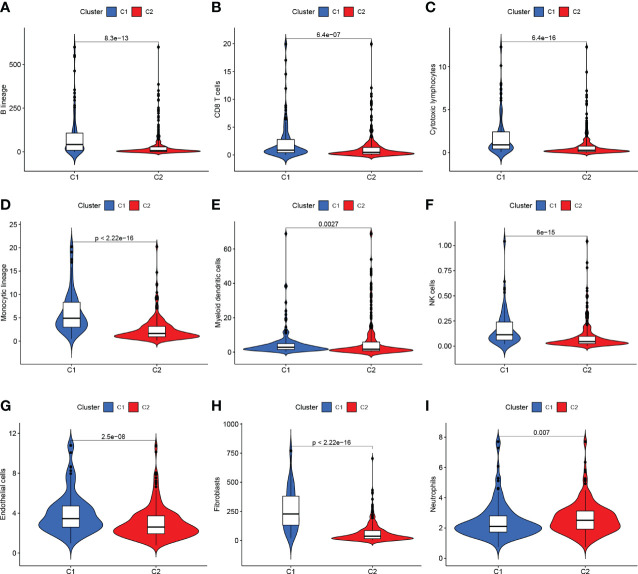
**(A–G)** C1 and C2 are different at the level of immune cells in the tumor microenvironment. **(H, I)** C1 and C2 are different at the mesenchymal cell level.

The TCGA-BLCA transcriptome data were clustered by NMF method. The stability and clustering performance were based on cophenetic and RSS. When K=2, clustering was the optimal value, that was, the sample can be divided into two categories, C1 and C2 (**Figures 1C, D**). The results of survival curve analysis show that the survival rate of C2 cluster samples was better than that of C1 cluster. (**Figure 1E**). It showed the relationship between the C1,C2 subtype and the classical immune subtype (Immune C1-C6) (**Figure 1F**). The distribution ratio between clusters of DEGs and the classical immune subtypes C1-C6 (**Figure 1G**). C1-C6 immune subtypes were: wound healing, IFN-gdominant, inflammatory, lymphocyte-depleted, immunologically quiet, and TGF-bdominant. The proportion of immune cell and stromal cell infiltration ratios of C1 and C2 was also significantly different (**Figures 2A–I**). Based on the above results, it was known that molecular subtypes based on gene clustering related to anoikis were associated with a variety of tumor microenvironment infiltrating cells.

### 3.3 Development and validation of prognostic prediction models for anoikis-associated genes

**Figure 3:**
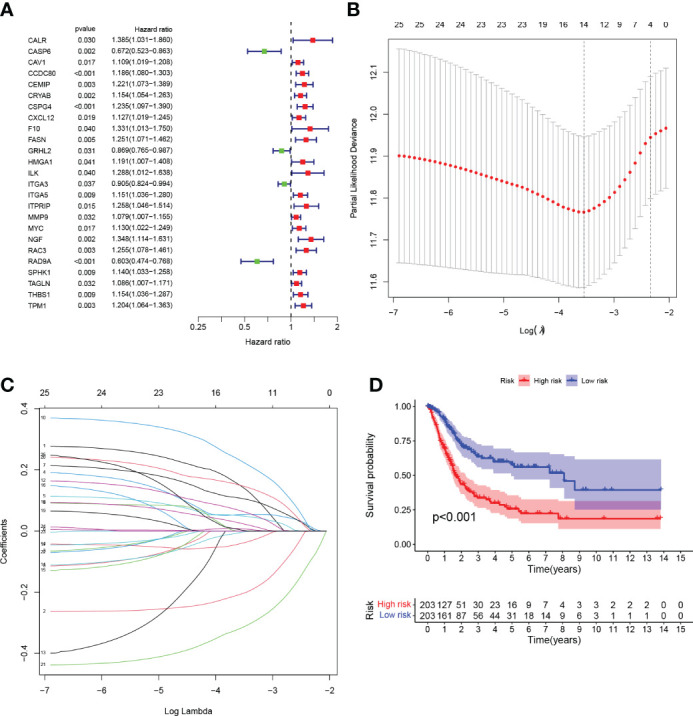
**(A)** Forest plot Univariate Cox regression analysis results of 25 anoikis-associated genes and overall survival. **(B)** LASSO coefficient profiles for 25 genes, marked with vertical lines at 10-fold cross-validation values. **(C)** Ten cross-validations for tuning parameter selection in lasso regression. Vertical lines are drawn based on the optimal data according to the minimum criterion and 1 standard error criterion. The left vertical line represents the 9 genes that were finally identified. **(D)** Kaplan-Meier curve analysis was performed on the TCGA database, showing the difference in overall survival between the HRG and LRG.

We performed univariate Cox regression analysis and identified 25 genes with significant prognostic value (P<0.05, **Table S3**; **Figure 3A**). To prevent overfitting, lasso regression and multivariate COX regression analysis were performed on these genes, and nine core genes (*CALR, CASP6, CCDC80, CSPG4, FASN, HMGA1, ITGA3, RAC3, RAD9A*) were identified in BLCA, all of which were considered prognostic indicators (**Table S4**; **Figures 3B, C**). RiskScore=(0.2879*CALR) - (0.3257*CASP6) + (0.0983*CCDC80) + (0.1480*CSPG4) + (0.3173*FASN) + (0.1698*HMGA1) - (0.0789*ITGA3) + (0.1792*RAC3) - (0.4413*RAD9A).

**Figure 4:**
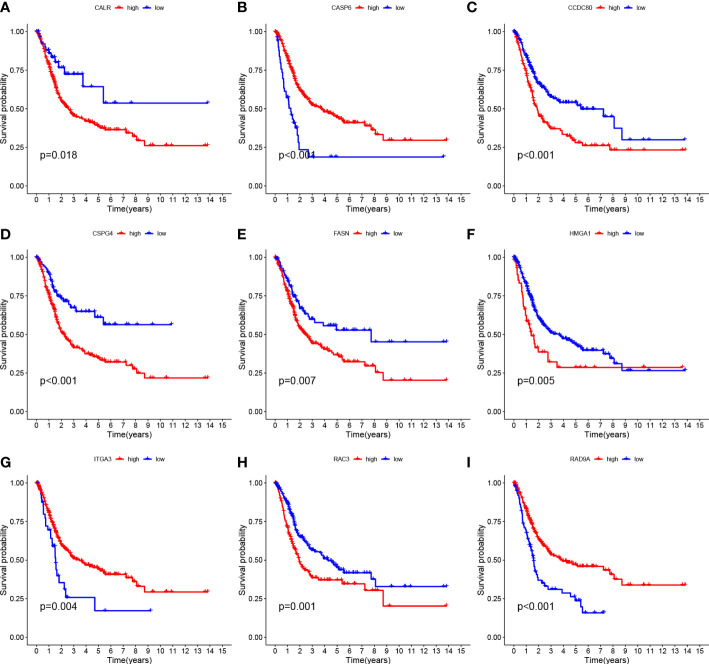
Survival curves of nine genes **(A–I)** *CALR*, *CASP6*, *CCDC 80*, *CSPG4*, *FASN*, *HMGA1*, *ITGA3*, *RAC3* and *RAD9A*.

Tumor samples were divided into HRG and LRG based on the median risk score. Survival analysis between HRG and LRG showed that the LRG had a better prognosis. (**Figure 3D**). In addition, survival analysis between subgroups with high and low expression of these nine core genes showed that abnormal mRNA expression of these genes also led to significant differences in overall survival time (P<0.05) (**Figures 4A–I**).

### 3.4 Constructing a risk nomogram

**Figure 5:**
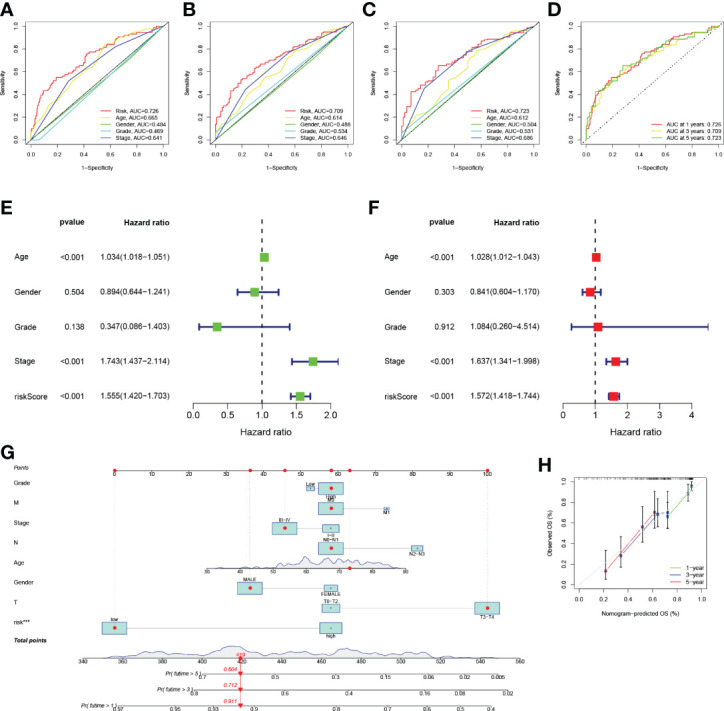
**(A–D)** ROC analysis predict 1-year, 3-year, and 5-year overall survival. **(E)** Results of univariate Cox regression analysis of overall survival. **(F)** Results of multivariate Cox regression analysis of overall survival. **(G)** Nomograph predicts survival. **(H)** Nomograph calibration curve.

The ROC curves were plotted, and the areas under ROC were 0.726, 0.709, and 0.723, respectively, indicating high prognostic validity (**Figure 5D**). Comprehensive analysis of risk score, age, sex, tumor grade, and clinicopathological stage over this 5-year period (**Figures 5A–C**) confirmed that the risk score had the highest area under the curve among different clinicopathological features, which could be used as a signal to monitor prognosis. Univariate, multivariate Cox regression results showed that age, stage, and risk score were independent prognostic factors in BLCA patients (**Figures 5E, F**). Based on the patient’s risk score and clinical variables, we constructed a nomogram to quantitatively predict a patient’s survival probability at 1, 3, and 5 years (**Figure 5G**). Using the nomogram, we can predict the patients survival. For example, a 55-year-old female patient was clinically diagnosed with BLCA, T3N0M1, and high-risk group. According to the model, the total nomogram score can be calculated as 419, and his survival probabilities at 1, 3, and 5 years are 91.1%, 71.2%, and 60.4%, respectively. The calibration curves showed that it has a good reliability (**Figure 5H**).

### 3.5 Functional analysis of anoikis-associated genes

**Figure 6:**
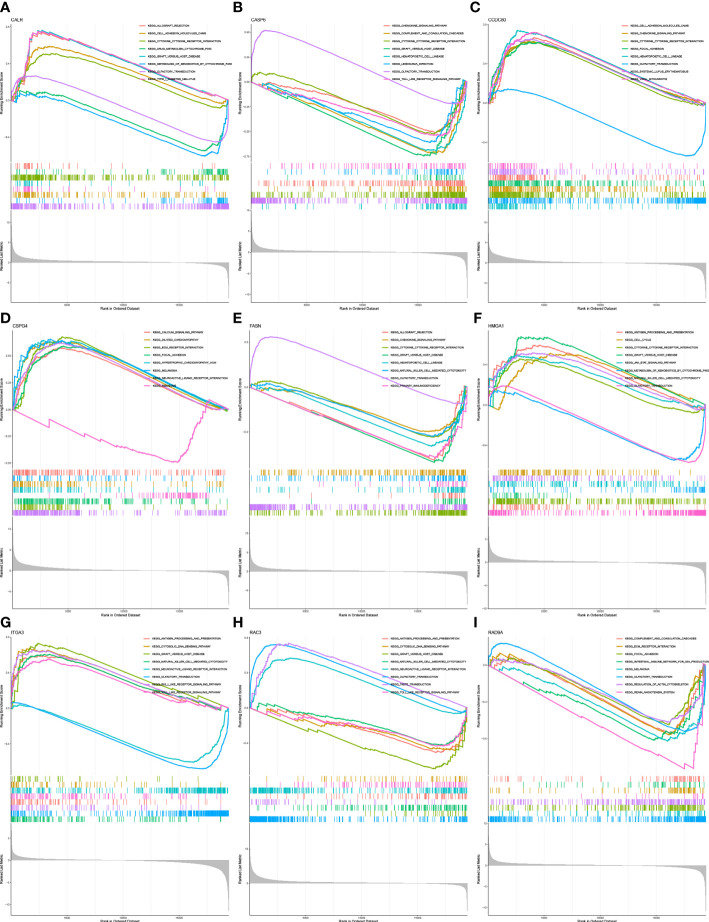
Functional enrichment **(A)** Enrichment gene set of a sample with high level expression of *CALR* in KEGG. **(B)** Enrichment gene set of *CASP6* high-level expression samples in KEGG. **(C)**: Enrichment gene set of *CCDC80* high-level expression samples in KEGG. **(D)** Enrichment gene set of *CSPG4* high-level expression samples in KEGG. **(E)** Enrichment gene set of samples with high level expression of *FASN* in KEGG. **(F)** Enrichment gene set of *HMGA1* high-level expression samples in KEGG. **(G)** Enrichment gene set of high-level *ITGA3* expression samples in KEGG. **(H)** Enrichment gene set of *RAC3* high-level expression samples in KEGG **(I)** Enriched gene set of *RAD9A* high-expression samples in KEGG.

GSEA was performed to identify the functional enrichment of high and low gene expression in 9 core genes. The KEGG enrichment term showed that the high expression of the *CALR* gene was related to Allograft-rejection, cell-adhesion-molecules, and other pathways. The activation of the Metabolism-of-xenobiotics-by-cytochrome pathway increases the expression of *CASP6* and *FASN* genes. The discovery of Graft-versus-host-disease and other signaling pathways is thought to be related to the high expression of *RAD9A* (**Figures 6A–I**).

### 3.6 Correlation of risk characteristics with clinicopathological variables

**Figure 7:**
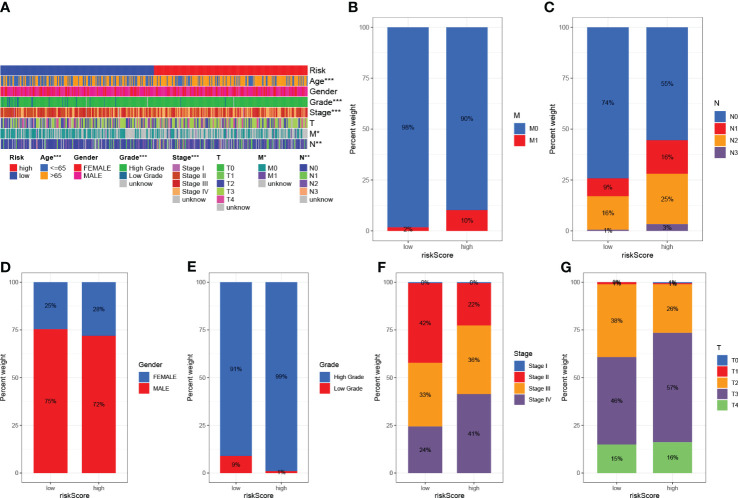
Clinical significance of predictive prognostic risk profiles. **(A)** The heat map shows the distribution of clinical features and the corresponding risk score in each sample. Incidence of clinical variable subtypes in the high and low risk score groups. **(B)** Distant metastasis M **(C)** Lymph node involvement N **(D)** Sex **(E)** Age **(F)** WHO grade **(G)** Tumor size. *P<0.05, **P<0.01, ***P<0.001.

The clinicopathological variables in the HRG and LRG were visualized (**Figure 7A**), and the proportional maps that could show the clinical variables in the HRG and LRG were, respectively, drawn (**Figures 7B–G**), and it was found that the HRG and LRG had obvious differences.

### 3.7 Clinical features are associated with tumor mutational burden

**Figure 8:**
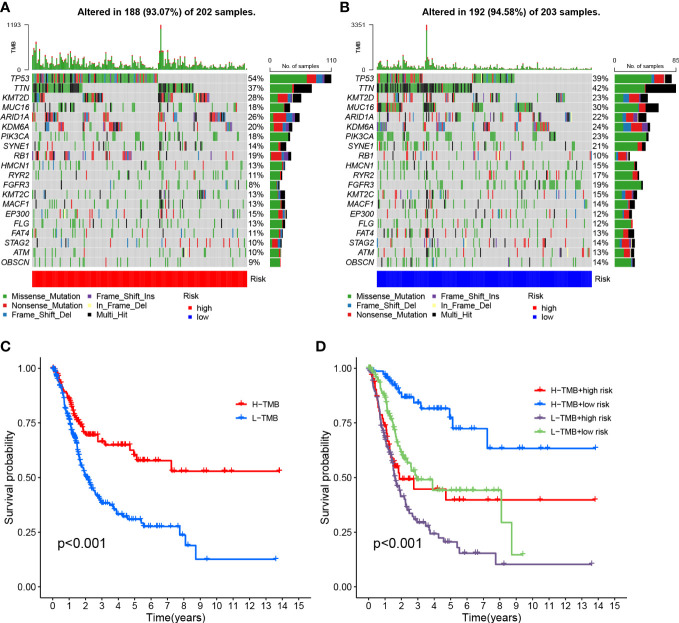
Risk score correlation with TMB **(A)** HRG creation oncoPrint **(B)** Low risk score creation oncoPrint **(C)** Kaplan-Meier curve for TMB high and low groups **(D)** Kaplan-Meier curve based on patients in TMB high and low risk score groups.

The survival curve indicates that the overall survival time for high TMB is longer (P<0.001, **Figure 8C**). Using TMB and risk scores, patients were divided into four groups, and the HRG and LRG showed significant prognostic differences in the high-TMB and low-TMB state subtypes (P<0.001, **Figure 8D**). This result proved that the risk score could predict the effectiveness of immunotherapy.

To investigate the correlation between risk scores and gene mutations, the top 20 gene waterfall plots that were most common among somatic mutations were shown (**Figures 8A, B**). The landscape of significant mutant gene (SMG) mutations showed that TP53 experienced a higher somatic mutation rate in the HRG (54% *vs* 39%), while FGFR3 had a higher somatic mutation rate in the LRG (19% *vs* 8%). These findings had the potential to benefit applications for anoikis in the treatment of BLCA.

### 3.8 Risk characteristics in the context of TIME

**Figure 9:**
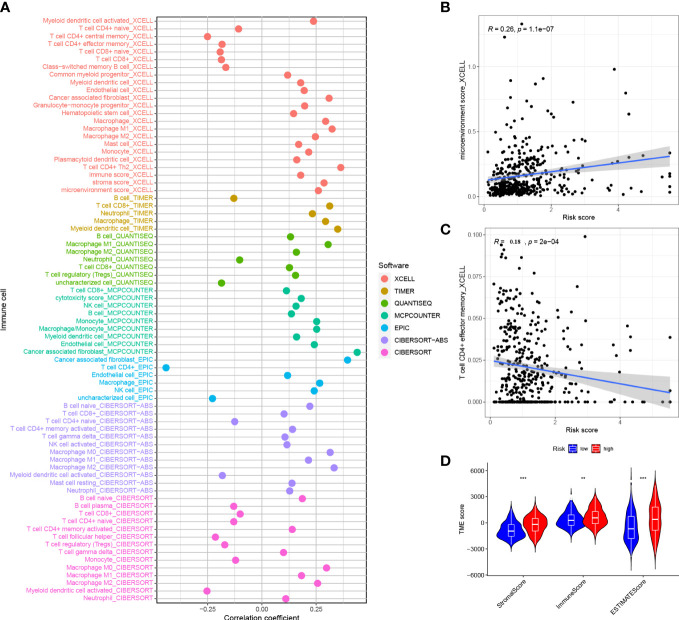
Correlation between risk score and immune invasion **(A)** Spearman analysis of the correlation between HRG patients and tumor infiltrating immune cells.**(B)** XCELL analyzed the relationship between infiltration and risk score of CD4+ T cells **(C)** XCELL analyzed the degree of invasion and risk score of immune microenvironment **(D)** ESTIMATE analyzed the TME score of the HRG and LRG. *P<0.05, **P<0.01, ***P<0.001.

The correlation of risk score and immune infiltration was further analyzed using seven different methods (**Figure 9A**). The results of EPIC analysis showed that the infiltration of CD4+ T cells was inversely proportional to the risk score, and the degree of infiltration of the immune microenvironment was directly proportional to the risk score (**Figures 9B, C**). The results of ESTIMATE analysis showed that there was a high trend in matrix scores and immune scores in both the HRG and LRG (**Figure 9D**).

### 3.9 Enrichment analysis of biological functions and signaling pathways

**Figure 10:**
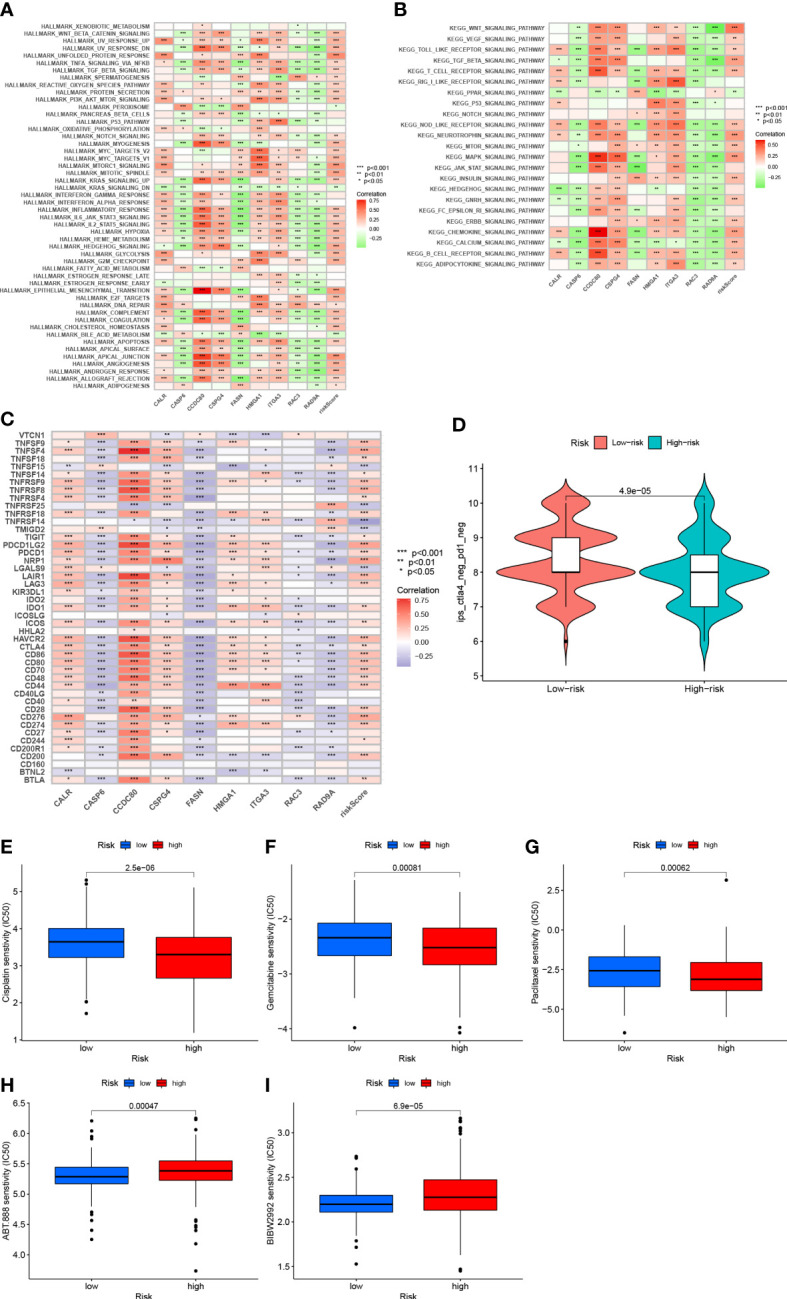
**(A)** Correlation between Hallmark’s representative pathway and risk score **(B)** Correlation between KEGG’s representative pathway and risk score. **(C)** Correlation between gene expression levels and risk scores at immune checkpoints. **(D)**: IPS score distribution chart **(E)** Sensitivity analysis of cisplatin in patients with high and low risk score groups **(F)** Sensitivity analysis of gemcitabine in patients with high and low risk score Group **(G)** Sensitivity analysis of paclitaxel in patients with high and low risk score **(H)** Sensitivity analysis of villipanib in patients with high and low risk score groups **(I)** Sensitivity analysis of afatinib in patients with high and low risk scores.

Gene set variation analysis of nine core genes further revealed the role of different risk groups in biology (**Figures 10A, B**). The results showed that the activity of PPAR signaling pathway was enhanced in subjects in the LRG, and the activity of signaling pathways such as WNT, BETA, MAPK, and other signaling pathways was enhanced in subjects in the HRG.

### 3.10 Prediction of patient immunotherapy outcomes

After further analysis, 47 genes related to checkpoint blockade were retrieved, and most of the checkpoints were negatively correlated with the risk score and were significant (**Figure 10C**). Predictive models showed low IPS scores in high-risk patients, indicating that high-risk patients may not be candidates for immunotherapy with PD-1 (**Figure 10D**). These results strongly suggest that risk scores correlate with response to immunotherapy and can be used to further predict prognosis.

### 3.11 Predicting chemotherapy response

Based on the pRRophetic algorithm, we evaluated the IC50 values of five chemotherapy drugs (paclitaxel, gemcitabine, cisplatin, velipanib, afatinib) in patients with BLCA. Paclitaxel, gemcitabine, and cisplatin showed higher IC50 (p<0.001) in the HRG, and the responsiveness to veliparib and afatinib in the LRG was better than that in the HRG (P<0.001) (**Figures 10D–I**). These results can provide appropriate recommendations for the use of chemotherapy drugs for patients with different risk scores.

### 3.12 The amount of expression of a gene in a cell

**Figure 11:**
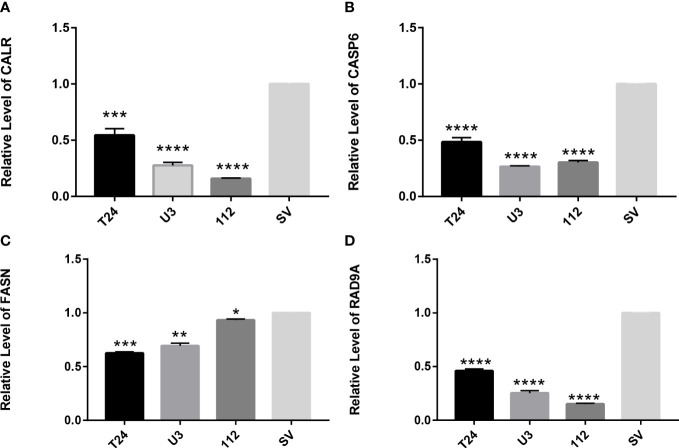
RT-qPCR verifies the expression of apoptosis-related genes in T24, UMUC3, RT112, and SV-HUC1 cells **(A)** Expression of *CALR* gene in cells **(B)** Expression of *CASP6* gene in cells **(C)** Expression of *FASN* gene in cells **(D)** expression of *RAD9A* gene in cells. *P<0.05, **P<0.01, ***P<0.001,****P<0.0001.

Four cell lines of T24, UMUC3, RT112, and SV-HUC1 were selected to verify the gene expression of *CALR*, *FASN*, *CASP6*, and *RAD9A*, respectively. The results showed that the gene expression of these three genes in cancer cells was significantly lower than that of normal cells, and the results were statistically significant. In summary, in cancer cells, anoikis-related genes were significantly inactivated, and these genes were somewhat associated with the progression of tumor cells (**Figure 11**).

## 4 Discussion

BLCA is a highly malignant urinary tumor, and genomic therapies such as regulation of noncoding RNAs, DNA methylation, and gene loci mutations are key regulators that prevent the continued progression of BLCA (14, 15). The regulation of these gene loci can not only inhibit the progression of cancer cells, but also promote cell death, which can optimize the treatment of BLCA to a certain extent.

Studies on the effects of the *CALR* gene have found that *CALR* is associated with immune responses and apoptosis of cells (16). There is more evidence that *CALR* is associated with cell carcinogenesis, drug resistance of tumor cells, and epithelial-mesenchymal transformation (17). The study found that *Casp6* in cancerous tissues was low expression, indicating that it had a certain inhibitory potential in tumorigenesis and progression (18). and the expression level of *Casp6* was inversely correlated with the IC50 value of 5fluorouracil (19, 20). *Casp6* is currently widely recognized as a key regulator of innate immuno-inflammatory activation and host defense (21). At present, it has been found that the increased expression of *CCDC80* can enhance the sensitivity of tumor cells to chemotherapy drugs, and knocking it down in cancer cells can enhance the anti-cancer drug resistance effect of bladder cancer cells (22). In the metabolic study of bladder cancer, it was found that the reduction of *FSAN* contributed to the metabolic conversion and proliferation of tumor cells (23). In studies of significant regulation of proteins related to DNA repair, including alterations in RAD9A, has been found to be associated with tumorigenesis, and similar conditions have been observed in B cells and other immune cells (24).

Clinical practice has shown that patients with high TMB are more conducive to immune checkpoint inhibitor therapy, TMB classified according to anoikis-associated genes in this study have a longer survival time (25). The function of the selected genes was enriched and analyzed, and the activity of signaling pathways such as WNT, BETA, and other signaling pathways in HRG subjects associated with anoikis was enhanced, and it was understood that the activation of these pathways promoted tumor survival and progression, and a variety of WNT and MAPK inhibitors could play a role in different cell cycles of aggressive bladder cancer cell growth (26). The enrichment of immune and carcinogenic pathways in the HRG and the high concentration of metabolism-related pathways in the LRG can explain the better prognosis and non-immunosuppressive state of the lower-risk group than in the HRG.

In short, anoikis plays a key role in the occurrence and development of tumors. Our study demonstrates the value of a group of anoikis-associated genes as biomarkers for BLCA prognosis. As a retrospective study conducted by bioinformatics analysis, the clinical application of this information is not perfect, and these biological mechanisms for predicting prognosis still need to be further confirmed.

## 5 Conclusion

In summary, we developed a model for predicting prognosis, immune microenvironment, and chemotherapy response in BLCA patients. The model was constructed based on nine anoikis-associated genes and clinical risk characteristics, and multidimensional validation of the model showed that the model has the potential to reliably predict clinical prognosis in BLCA species.

## Data availability statement

The original contributions presented in the study are included in the article/**Supplementary Material**. Further inquiries can be directed to the corresponding author.

## Author contributions

CX, YD, GS and HM designed this work. YD, CX, YaL, BY, YuL, GS, TY, SM, HM contributed to data analysis. YD and CX wrote this manuscript. HM edited and revised the manuscript. All authors contributed to the article and approved the submitted version.

## Supplementary material

Supplementary Table 1 The anoikis -related genes from GeneCards database.

Supplementary Table 2 434 DEGs between BLCA tissue and normal bladder tissue.

Supplementary Table 3 The results of univariate regression analysis.

Supplementary Table 4 The results of multivariate regression analysis.
